# Evaluating sub-typing methods for pathogenic *Yersinia enterocolitica* to support outbreak investigations in New Zealand

**DOI:** 10.1017/S0950268819000773

**Published:** 2019-04-30

**Authors:** H. Strydom, J. Wang, S. Paine, K. Dyet, K. Cullen, J. Wright

**Affiliations:** Institute of Environmental Science & Research (ESR), Porirua, New Zealand

**Keywords:** Outbreaks, surveillance, *Yersinia enterocolitica*

## Abstract

Incidence of human yersiniosis in New Zealand has increased between 2013 and 2017. For surveillance and outbreak investigations it is essential that an appropriate level of discrimination between pathogenic *Yersinia enterocolitica* isolates is provided, in order to support epidemiological linking of connected cases. Subtyping of 227 *Y. enterocolitica* isolates was performed using a range of different typing methods, including biotyping, serotyping and seven loci multiple-locus variable-number tandem-repeat analysis (MLVA). In addition, core genome single-nucleotide polymorphism (core SNP) analysis and multi-locus sequence typing were performed on a subset of 69 isolates. Sixty-seven different MLVA types were identified. One MLVA profile was associated with an outbreak in the Bay of Plenty region, supported by epidemiological data. Core SNP analysis showed that all the outbreak-related isolates clustered together. The subtyping and epidemiological evidence suggests that the outbreak of yersiniosis in the Bay of Plenty region between October and December 2016 could be attributed to a point source. However, subtyping results further suggest that the same clone was isolated from several regions between August 2016 and March 2017. Core SNP analysis and MLVA typing failed to differentiate between *Y. enterocolitica* biotype 2 and biotype 3. For this reason, we propose that these biotypes should be reported as a single type namely: *Y. enterocolitica* biotype 2/3 and that the serotype should be prioritised as an indicator of prevalence.

## Introduction

New Zealand has a high rate of yersiniosis notifications compared with other developed countries and this rate is increasing [1]. Between 2013 and 2017 the yersiniosis notification rate has nearly doubled, increasing from 10.8 [2] to 19.2 [3] per 100 000 population. By comparison, the European Union (EU) reported a notification rate of 1.7 cases per 100 000 population in 2017 with no significant increase or decrease between 2013 and 2017 [4]. In New Zealand a confirmed case requires laboratory evidence, including isolation of *Yersinia enterocolitica* or *Yersinia pseudotuberculosis* from blood or faeces or detection of *Yersinia* species nucleic acid from faeces [5]. The case definition for yersiniosis is comparable between New Zealand and the EU with the exception of a requirement to detect virulence factors in the EU [5, 6]. In addition, the absence of mandatory surveillance in some EU member states may contribute to lower notification rates. The increased notification rate in New Zealand could be due to factors such as better awareness and more susceptible analytical techniques; however, the more likely cause is an increased burden of disease. Irrespective of the cause of this increase it necessitates better laboratory techniques to support epidemiological investigation.

Yersiniosis is primarily a foodborne disease caused by the enteropathogenic bacteria *Y. enterocolitica* and *Y. pseudotuberculosis* [7]. Typical yersiniosis symptoms range from self-limiting gastroenteritis to terminal ileitis and mesenteric lymphadenitis. The right lower quadrant location of the resulting abdominal pain can be mistaken for appendicitis. Potential post-infectious sequelae include arthritis and erythema nodosum [8, 9]. It is thought that almost 75% of *Y. enterocolitica* cases reported in New Zealand are due to foodborne transmission with more than 50% of those being due to pork [10]. Identifying the source of yersinia infection has previously been hampered by low bacterial cell numbers when testing food and environmental samples [11].

*Y. enterocolitica* is a heterogeneous species that can be divided into two sub species: *Y. enterocolitica* subsp. *enterocolitica* and *Y. enterocolitica* subsp. *palaerctica* [12]. *Y. enterocolitica* has traditionally been characterised using biotyping based on biochemical reactions [13, 14] and serotyping [15]. There are six recognised *Y. enterocolitica* biotypes (1A, 1B, 2, 3, 4 and 5) [16]. Interpreting biotyping reactions can be subjective and misidentification of *Y. enterocolitica* biotypes is common [16]. *Y. enterocolitica* subsp. *enterocolitica* is biotype 1B and considered to be highly virulent. *Y. enterocolitica* subsp. *palaerctica* consists of biotypes 1A, 2, 3, 4 and 5 [14]. *Y. enterocolitica* subsp. *palaerctica* is hereafter called *Y. enterocolitica* unless otherwise stated. Biotypes 2, 3, 4 and 5 are considered pathogenic with biotypes 2, 3 and 4 being the most common causes of human gastrointestinal yersiniosis globally [13]. Some consider *Y. enterocolitica* biotype 1A non-pathogenic due to the lack of major virulence factors such as the plasmid pYV; invasin, YadA and Ail [15]. However, it has been suggested that some *Y. enterocolitica* biotype 1A strains may cause human disease using alternative mechanisms [7, 17]. For this reason, it is important to differentiate biotype 1A from other biotypes. *Y. enterocolitica* biotype 1A infection meets the case definition for notification in New Zealand.

The recent increase of yersiniosis in New Zealand can be largely attributed to an increase in *Y. enterocolitica* biotype 2 [5]. Since 2014, *Y. enterocolitica* biotype 2 has emerged as the most common biotype causing yersiniosis in New Zealand, surpassing *Y. enterocolitica* biotype 4. The hospitalisation rate associated with this biotype has increased proportionally to the notification rate (data not shown), suggesting that the pathogenic effect has not substantially altered.

There are more than 70 *Y. enterocolitica* serotypes based on differences in surface antigens [14]. Only 11 serotypes are known to cause yersiniosis [15]. The serotypes most frequently associated with human disease are O3; O8; O9 and O5,27. O3 is the most commonly identified serotype world-wide [18].

Typing of yersinia is critical for effective surveillance, outbreak investigation and source attribution studies. An ideal typing system should link bacterial isolates related to the same source and exclude non-related isolates. Neither biotyping nor serotyping provides sufficient discrimination for such purposes [19].

Pulse-field gel electrophoresis (PFGE) has been used successfully by our group and others to investigate outbreaks of other foodborne diseases [8, 20–22]. PFGE appears to have sufficient discriminatory power when typing biotype 1A [8]. However, a lack of discriminatory power has been found when assessing *Y. enterocolitica* biotypes 2, 3 and 4 [8, 23]. Lack of diversity between these biotypes can in part be attributed to the relatively high number of non-cutting patterns when using PFGE [8]. In addition, some enzymes used for subtyping *Y enterocolitica* produce very closely spaced restriction fragments, which can further complicate analysis [24]. For biotypes 2, 3 and 4, PFGE profiles of isolates associated with outbreaks may be indistinguishable from those of sporadic isolates, potentially leading to erroneous conclusions about the relationships among cases. Because of this high level of homogeneity of PFGE profiles we did not include PFGE in this study but instead investigated other typing methods.

Variable-number tandem repeats (VNTR) are short DNA sequences repeated a number of times in tandem. Variability in the number of repeats is caused by mechanisms such as slippage and mispairing during DNA replication. This variation in alleles can be identified using polymerase chain reaction (PCR) by amplifying the repeats as well as the flanking regions and determining the size of the amplicon [18]. During the last decade, a number of multiple-locus variable-number tandem-repeat analysis (MLVA) assays have been developed for *Y. enterocolitica* [18, 25]. MLVA utilises variation in multiple (VNTR) loci to differentiate between isolates. This method offers better discriminatory potential than PFGE [18]. In this study, we employed a scheme that uses seven primer sets encompassing regions of the *Y. enterocolitica* genome that contain VNTR sequences.

In recent years, the cost of whole-genome sequencing (WGS) has decreased allowing it to be used for public health surveillance and outbreak investigation [26, 27]. To the best of our knowledge, there is no report on the use of WGS of *Y. enterocolitica* in an outbreak investigation in New Zealand. However, Williamson *et al*. [28] have used WGS to investigate an outbreak of *Y. pseudotuberculosi*s.

This study describes various methods used for subtyping *Y. enterocolitica*, including biotyping, serotyping, MLVA, multi-locus sequence typing (MLST) and core genome single-nucleotide polymorphism (core SNP) analysis. More importantly, this study aims to determine what sub-typing method(s) offers the best level of discrimination; and tests the ability of subtyping methods to cluster *Y. enterocolitica* isolates from epidemiologically linked cases. An appropriate level of discrimination is required to assist in surveillance activities and outbreak investigations.

## Methods

### Bacterial isolates

Diagnostic laboratories serving District Health Boards (DHBs) throughout New Zealand refer yersinia isolates to the Enteric Reference Laboratory (ERL) at the Institute of Environmental Science and Research (ESR) for confirmation and further testing. Following identification and biotyping isolates are maintained on Dorset egg slopes. Biotyping was performed according to the scheme described by Petersen *et al*. [29] and consisted of the following biochemical reactions: fermentation of xylose, trehalose and salicin; production of indole and lipase; and hydrolysis of aesculin.

For MLVA analysis we selected a representative subset from isolates referred to ERL between August 2015 and March 2017. We included isolates from both sporadic (*n* = 207) and epidemiologically linked cases (*n* = 20). Of those 213 were *Y. enterocolitica* biotype 2, 10 were biotype 3, and two were biotype 4. These isolates were sub-cultured on trypticase soy agar (Lab M, Lancashire, UK) and incubated at 28 °C for 18 h. Serotyping was performed using *Y. enterocolitica* antisera for O3, O5, O8 and O9 (SSI Diagnostica, Hillerød, Denmark) and O27 (SIFIN, Berlin, Germany). Genomic DNA was extracted using a DNeasy tissue kit (Qiagen, Hilden, Germany) as per the manufacturer's instructions for Gram-negative bacteria and eluted in either 100 µl AE buffer for PCR or eluted in 100 µl 10 mM Tris pH 8.0 for WGS.

### MLVA PCR and analysis

MLVA PCR was performed using two multiplex reactions with the Type-It Microsatellite PCR reagent (Qiagen, Hilden, Germany), 15 µl each. Seven previously described VNTR loci were amplified namely: VNTR1 and VNTR3 [25] as well as V6, V9, V2a, V5 and V7 [18]. All forward primers for each VNTR loci contained a fluorescent dye as 5′ modification. VNTR1, V9 and V2a were labelled with FAM; VNTR3 and V7 were labelled with HEX; and V5 and V6 were labelled with CAL Fluor Red 590. Primers were synthesised by Biosearch Technologies (Petaluma, USA). Primer concentrations were 0.13 µM (VNTR1), 0.13 µM (VNTR3) and 0.33 µM (V6) in the first PCR reaction and 0.27 µM (V9), 0.13 µM (V2a), 0.33 µM (V5) and 0.33 µM (V7) in the second PCR reaction. Amplification was performed using a ABI ProFlex PCR System (Applied Biosystems, Foster City, USA) and the following cycling conditions: initial denaturation at 95 °C for 5 min; followed by 35 cycles each of denaturation at 95 °C for 30 s, annealing 57 °C for 90 s and extension at 72 °C for 45 s, and a final extension at 72 °C for 5 min.

PCR products were diluted 1:100 in DNase/RNase free water and submitted to the Waikato DNA Sequencing Facility (Hamilton, New Zealand) for genotyping. Capillary electrophoresis was performed using a 3130 Genetic Analyzer (Applied Biosystems). GeneScan™ 600 LIZ^®^ (Applied Biosystems) was used as a size standard and data were analysed using GeneMapper^®^ Software 5 (Applied Biosystems). MLVA types were denoted as a string of numbers representing the size of products (Table S1 in the supplemental material) from each of the seven loci in the following order: VNTR1-VNTR3-V6-V9-V2a-V7-V5, e.g. 4-10-5-3-3-6-10 and then subjected to cluster analysis. A minimum spanning tree was generated to cluster similar MLVA types using BioNumerics software (version 7.6, Applied Maths, Kortrijk, Belgium). Single locus VNTR allelic variables were partitioned together. Simpson's discriminatory indexes (SDI) were calculated according to Hunter and Gaston [30] using the Comparing Partitions website [31].

### Whole-gene sequencing and bioinformatics analysis

The whole genomes of a subset of isolates selected for MLVA typing were sequenced. We included isolates from sporadic (*n* = 52) as well as epidemiologically linked (*n* = 17) cases. Of those 57 were *Y. enterocolitica* biotype 2, 10 were biotype 3 and two were biotype 4 isolates.

The DNA library was created using the Nextera XT DNA preparation and sequencing was performed using an Illumina MiSeq platform. Sequencing quality assessments were performed using the Nullarbor version 1.20 pipeline [32]. Raw reads were trimmed using Trimmomatic version 0.36 [33]. MLST sequence type (ST) assignment was performed using MLST version 2.6 with the seven loci McNally scheme described in [16]. Core SNP analysis was performed using Snippy version 3.1 [34] and SnapperDB 1.4 [35] with *Y. enterocolitica* (type O9) str. YE212/02 as the reference genome. A cluster was defined as less than or equal to five SNP differences. A maximum likelihood tree was inferred on the 12 819 core SNP alignment using FastTree [36] with 1000 bootstraps and visualised using Phandango [37].

### Epidemiological surveillance

Surveillance of clinical yersinia infections in New Zealand is achieved through laboratory and public health collaboration. As yersiniosis is a notifiable disease, general practitioners and laboratories are required to notify all cases of *Y. enterocolitica* and *Y. pseudotuberculosis* to a Medical Officer of Health. Case information is recorded on EpiSurv, the national notifiable disease database.

An extended questionnaire covering a wide range of potential risk factors was developed for hypothesis generation and was applied to 104 yersiniosis cases reported from October 2015 to November 2016. Isolates from 46 of these cases were MLVA typed. Information on the cases' activities in the week prior to symptom onset was collected and included details not limited to contact with water, animals and the environment, other sick people; travel, diet, lifestyle, food-shopping habits and food and water consumed at or away from home.

Data from extended questionnaires was collected and managed using REDCap (Research Electronic Data Capture) electronic data capture tools hosted at ESR [38]. Data management and calculation of summary statistics was performed using Stata 14.0 (StrataCorp LLC, Texas) and Excel 2013 (Microsoft).

## Results

**Fig. 1.**
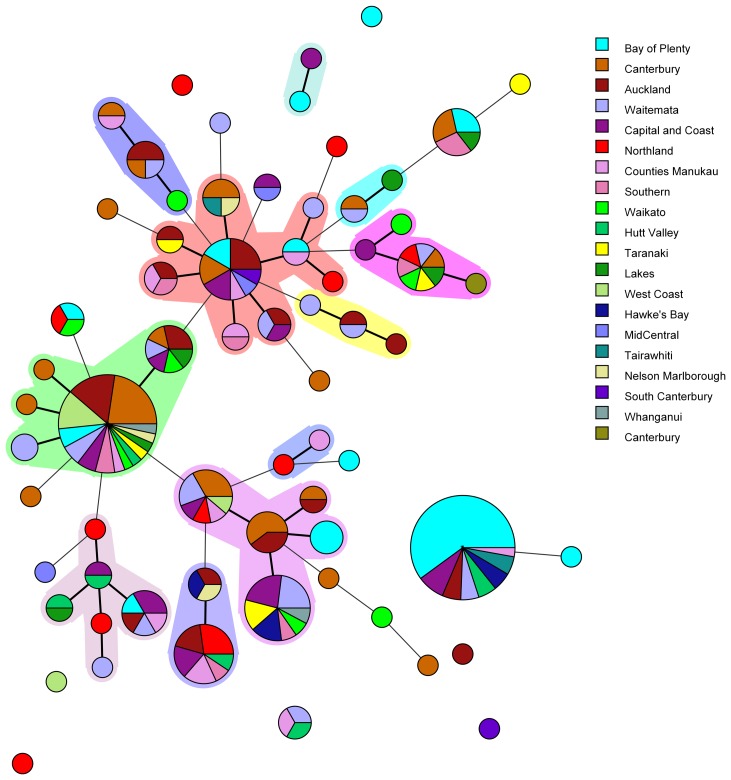
Minimum spanning tree of 227 pathogenic *Y. enterocolitica* generated with BioNumerics v 7.6. Each circle represents a MLVA profile and its size is proportional to the number of isolates. Each colour represents a different geographical region. Partitioning grouped MLVA profiles that vary in one or less loci. Branches link those MLVA profiles that vary in two or less loci.

Amongst the 227 isolates tested 67 different MLVA profiles were identified (Fig. 1). Multiple alleles were identified for each locus namely: VNTR1 (9), VNTR3 (9), V6 (7), V9 (4), V2a (4), V7 (9) and V5 (9). Five MLVA profiles accounted for 45% of isolates: 4-10-5-3-3-6-10 (*n* = 35), 4-7-8-4-4-6-7 (*n* = 31), 4-8-7-4-4-6-8 (*n* = 13), 5-7-7-4-4-7-7 (*n* = 12) and 3-8-7-4-4-5-8 (*n* = 11). All five of these MLVA profiles were identified for cases from multiple regions (see Table S2 in the supplemental material). The SDI for sporadic isolates was 0.960 for MLVA.

The most common MLVA profile, 4-10-5-3-3-6-10, was isolated from patients in the following DHBs: Bay of Plenty (*n* = 21), Capital and Coast (*n* = 3), Hawke's Bay (*n* = 2), Hutt Valley (*n* = 2), Waitemata (*n* = 2), Auckland (*n* = 2), Tairawhiti (*n* = 2) and Counties Manukau (*n* = 1). All the Bay of Plenty cases were reported between October 2016 and March 2017.

Epidemiological data identified an outbreak of 24 cases of yersiniosis in the Bay of Plenty between October and December 2016, that were linked to three food premises serving sushi [39]. A total of 21 isolates were available from the cases; 19 were *Y. enterocolitica* biotype 2 and two were *Y. enterocolitica* biotype 3. Twenty of the isolates epidemiologically linked to this outbreak were characterised using MLVA. Isolates obtained from 19 cases who had eaten from the implicated premises had MLVA profile 4-10-5-3-3-6-10 in common and one had a similar profile: 4-11-5-3-3-6-11, that differed at two loci. As a result, the outbreak definition was expanded to include these MLVA profiles. Thirteen cases from DHBs outside the Bay of Plenty, with the same MLVA profile 4-10-5-3-3-6-10, were also reported between August 2016 and March 2017. An extended questionnaire revealed that one of the cases reported outside of the Bay of Plenty region, with the same MLVA profile as the outbreak, had eaten sushi from an unidentified source, but it is unknown whether other cases that shared this MLVA profile had also eaten sushi.

Another common MLVA profile 4-7-8-4-4-6-7 was identified in isolates from 31 cases, from 12 different DHBs, notified between November 2015 and December 2016. However, no epidemiological association was found to link these cases and therefore consider it an outbreak of yersiniosis. Among this group 13/31 (42%) identified as being Asian (Chinese (4), Filipino (3), Other Asian (not further defined) (2), Indian, Japanese, Cambodian, Southeast Asian (not elsewhere classified) (1 each)). Approximately 12% of the New Zealand population identify as being Asian [40].

WGS data for 69 isolates tested had a minimum coverage of 45 and an average quality score above 32, which have been considered as suitable quality parameters for downstream analysis [32]. Isolates selected consisted of 57 *Y. enterocolitica* biotype 2, 10 biotype 3 and two biotype 4 (Table S2). It included sporadic and outbreak strains. MLST STs were as follows: ST12 (*n* = 61), ST14 (*n* = 6) and ST18 (*n* = 2). All ST12 isolates were serotype O:9 comprising seven biotype 3 and the remainder biotype 2. All ST14 were serotype O:5, 27 comprising three biotype 3 and three biotype 2. All ST18 were serotype O:3, biotype 4. ST12, ST14 and ST18 clustered separately when analysed using core SNP analysis. However, biotype 2 and biotype 3 (but not biotype 4) clustered together. The SDI for sporadic isolates was 0.956 for core SNP analysis.

In the ST12 cluster five major sub-clades were present. One of which had less than five SNP differences and contained all of the isolates with Bay of Plenty outbreak MLVA profile 4-10-5-3-3-6-10 of which some were biotype 2 and some were biotype 3.

## Discussion

Biotyping and serotyping have historically been used to differentiate between isolates beyond the species level. *Y. enterocolitica* isolates that have the same biotype or serotype are more likely to be related to each other than if they had different biotypes or serotypes. Therefore, these subtyping methods are a useful starting point for public health surveillance purposes. Incidence of yersiniosis that is caused by a particular biotype and serotype at levels higher than expected may indicate an outbreak and will require further investigation. To assist public health teams with their outbreak investigations it is essential that subtyping provides an appropriate level of discrimination to link epidemiologically associated cases. However, biotyping and serotyping alone do not offer an adequate level of discrimination and are unable to link bacterial isolates to the same source while excluding non-related isolates. In addition, our results show that biotypes 2 and 3 cluster together by MLVA and core SNP analysis and epidemiologically linked cases would have been excluded from the outbreak if biotype results had been incorporated in the case definition. Therefore, additional subtyping is needed. In addition to having high discriminatory power a good typing system for routine epidemiological surveillance and investigation should be generally available and inexpensive [41, 42]. Previously genetic homogeneity for *Y. enterocolitica* biotypes 2, 3 and 4 using PFGE was reported [8, 23]. A further drawback for PFGE is that it is labour intensive and time-consuming.

MLVA has previously been used for subtyping of yersinia [18, 25, 43, 44]. Gierczynski *et al*. [18] were able to identify 76 MLVA profiles among 91 isolates of *Y. enterocolitica*. Previous studies have shown that MLVA has a higher discriminatory power than PFGE. An SDI for sporadic strains was determined in a previous study to be 0.999 for MLVA and 0.862 for PFGE [44, 45]. In the current study, the SDI for the sporadic isolates was 0.960 for MLVA and 0.956 for core SNP analysis. A typing system should at least have an SDI of 0.950 to be considered ideal [41]. For the purpose of this study we have decided to exclude the VNTR loci V4 as described by Gierczynski *et al*. [18] due to a low reported discriminatory power [43–45]. Instead we included VNTR1 and VNRT3 described by Gulati *et al*. [25]. In addition we included VNTR loci V6, V9, V2A, V7 and V5 for which the discriminatory power in a previous study has been 84.9%, 46.0%, 91.1%, 82.1% and 83.3%, respectively [44]. In the current study the discriminatory index for loci VNTR1, VNTR3, V6, V9, V2a, V7 and V5 was 66.6%, 70.8%, 71.0%, 41.3%, 30.4%, 66.6% and 70.8%, respectively. Sihvonen *et al*. showed V2A to have the highest discriminatory power by resolving the highest number of alleles (*n* = 17) [45], whereas our study resolved the least number of alleles (*n* = 4) for this locus. With regards to such discrepancy Virtanen *et al*. argued that regional differences in discriminatory power for a specific locus do exist [44]. Similar to our study, lower discriminatory ability for V2A has been noted among Chinese strains, especially for *Y. enterocolitica* biotype 2 isolated in the Ningxia province [43].

In the current study MLVA showed potential for epidemiological investigation of *Y. enterocolitica* in New Zealand during outbreak investigations, as we were able to identify 67 MLVA profiles among 227 isolates investigated. Five common MLVA profiles were identified accounting for 45% of isolates tested. All common MLVA profiles were from yersiniosis cases in multiple geographical regions. MLVA profile 4-10-5-3-3-6-10 and related profile 4-11-5-3-3-6-11 were epidemiologically linked during an ongoing outbreak investigation in the Bay of Plenty region. This allelic variation between epidemiologically linked MLVA profiles support previous studies suggesting that variation in several VNTR loci is not uncommon in isolates assumed to belong to the same strain [46]. It has previously been reported that both insertions and deletions that led to a different MLVA profile were observed from a strain isolated from piglets originating from the same farm [46]. It was assumed that the same strain persists on a single farm. Although such variable loci may be over-discriminatory and unlikely to provide useful information for routine long term surveillance, our study has shown that MLVA offers an appropriate level of discrimination to allow clustering of epidemiologically associated cases during short-term studies such as outbreak investigations.

Noller *et al*. [47] suggest that during outbreak situations epidemiologically linked cases with indistinguishable MLVA profiles should be considered to have originated from the same source, while a single or double tandem repeat difference between isolates at a single VNRT locus should also be considered to have originated from a point source [47]. The current study found that inferring a genetic relationship between isolates showing allelic variation in multiple VNTR loci without epidemiological association should be avoided as it requires a better understanding of the clonal nature of each locus. This finding is in agreement with Van Belkum *et al*. who argued that a major drawback of MLVA typing is that the evolution of repetitive DNA may be too rapid [41]. A higher degree of stability for individual VNTR loci is required for a typing method to be used for routine surveillance over longer time periods. Considerations should include geographical differences in discriminatory power among different loci as well as environmental impact on the stability of tandem repeats. Most importantly, cases considered linked based on MLVA profiles should be supported by epidemiological evidence.

MLVA offered a level of discrimination able to cluster isolates related to the same outbreak while excluding non-related isolates. In addition, our study found MLVA typing to be less time consuming and labour intensive than PFGE while still being inexpensive and relatively accessible. Concordance between MLVA typing and epidemiological evidence allowed for the outbreak definition to be expanded to include the MLVA type and for sporadic cases to then be excluded. By contrast we are unable to elucidate the significance of the 31 isolates from 12 different regions which shared the MLVA profile 4-7-8-4-4-6-7 but lacked an epidemiological association.

**Fig. 2.**
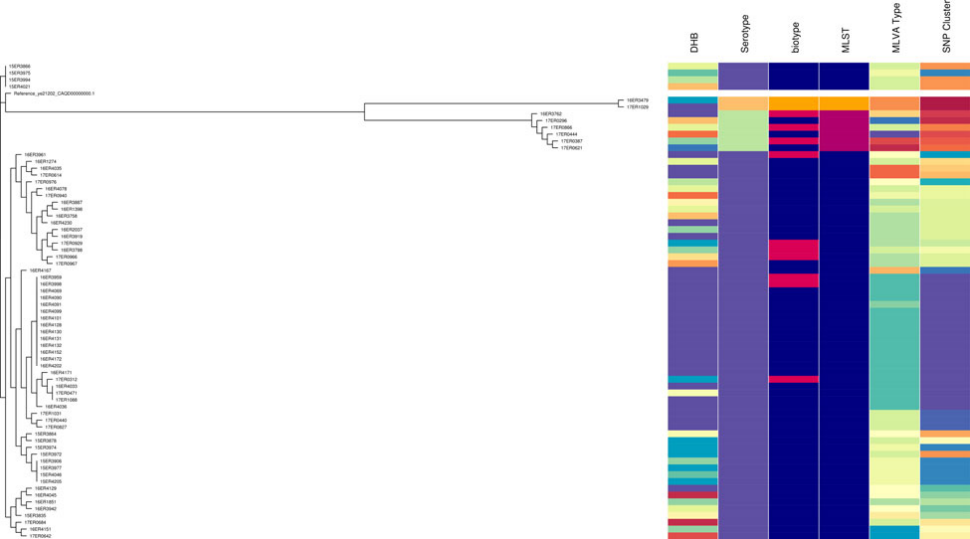
Core SNP analysis performed using Snippy version 3.1 and SnapperDB 1.4. A maximum likelihood tree was inferred on the 12 819 core SNP alignment using FastTree and visualised in conjunction with the corresponding DHB, serotype, biotype, MLST type, MLVA profile and core SNP cluster as well as using Phandango (these data should be interpreted in conjunction with Fig. S2).

Like MLVA typing, core SNP analysis offered a high level of resolution (Fig. 2) and supported epidemiological evidence that the Bay of Plenty outbreak of yersiniosis during October and November 2016 [39] may be attributed to a point source. Both these subtyping methods indicate that the same clone was isolated from several cases in multiple regions between August 2016 and March 2017 (Fig. 1, temporal data not shown). Furthermore, Both MLVA and core SNP analysis clustered *Y. enterocolitica* biotype 2 and biotype 3 together, but separate from biotype 4 (Fig. 2). This was also observed by Reuters *et al*. [48]. Subsequently these isolates were retested. Some isolates previously identified as biotype 2 were shown to be biotype 3 and vice versa. The discriminating test, a delayed weak indole reaction, can be subjective and our results support findings by Hall *et al*. [16]. For this reason, we question the continued description of *Y. enterocolitica* biotype 2 and *Y. enterocolitica* biotype 3 as two different epidemiological entities.

MLST results concurred with serotype results. However, like serotyping the level of discrimination provided by MLST is very limited, inadequate for thorough surveillance purposes and unsuitable for outbreak investigations.

People identifying as Asian were disproportionately represented in our data and this requires further investigation. An extended questionnaire showed that cases were exposed to a wide range of food, animal and environmental sources (data not shown). No obvious exposures, with the exception of cases linked to the Bay of Plenty outbreak, were determined from the case analysis. Pork consumption was investigated as this has previously been associated with yersiniosis in New Zealand [49]. Two-thirds (68.4%, 39/57) of cases identified having eaten pork and over a third (36.8%) of those had handled raw pork. It is not known what the population baseline of pork consumption was during this time.

## Conclusion

This study was based on testing sporadic and outbreak-related isolates received from diagnostic laboratories throughout New Zealand between August 2015 and March 2017 and linking this typing information with epidemiological data collected from the cases.

We used serotyping, seven-loci MLST, core SNP analysis and MLVA typing to further differentiate between isolates predominantly belonging to biotype 2 and biotype 3. Core SNP analysis and MLVA typing failed to differentiate between *Y. enterocolitica* biotype 2 and biotype 3. For this reason, we propose that these biotypes should be reported as a single type namely: *Y. enterocolitica* biotype 2/3 and that the serotype should be prioritised as an indicator of prevalence.

MLVA and core SNP analysis offer greater discrimination than MLST, biotyping, serotyping and PFGE. They provide accurate case connection within a reasonable timeframe and budget. For both MLVA typing and core SNP analysis we demonstrated an acceptable discriminatory power and both methods were able to cluster together epidemiologically associated isolates.

Good communication between laboratories, bioinformaticians, epidemiologists and local case investigators is essential to ensure timely recognition of linked cases and subsequent targeted investigations. Further studies to better understand sources and transmission pathways of yersinia in the New Zealand context are required.

## Supplementary material

For supplementary material accompanying this paper visit https://doi.org/10.1017/S0950268819000773. click here to view supplementary material
