# Supplementary material for: Evaluating sub-typing methods for pathogenic Yersinia enterocolitica to support outbreak investigations in New Zealand
Source: Epidemiol Infect. 2019 Apr 30;147:e186. doi: 10.1017/S0950268819000773 (PMC6518587; doi:10.1017/S0950268819000773)
Supplement: Supplementary file 1 [file S0950268819000773sup001.docx]

*Epidemiology and Infection*

Evaluating sub-typing methods for pathogenic Yersinia enterocolitica to support outbreak investigations in New Zealand.

H. STRYDOM, J. WANG, S. PAINE, K. DYET, K. CULLEN and J. WRIGHT

correspondence to: [Jackie.wright@esr.cri.nz](mailto:Jackie.wright@esr.cri.nz)

Supplementary Material

**This file includes:**

Tables S1 and S2

Table S1.

Assignment of allele nomenclature presenting the size of products from each of the seven loci. A maximum tolerance of 2 bp has been allowed for each loci with the exception of V9 for which 3 bp has been allowed.

| **Allelic nomenclature** | **VNTR1** | **VNTR3** | **V6** | **V9** | **V2a** | **V7** | **V5** |
| --- | --- | --- | --- | --- | --- | --- | --- |
|  | **Size of PCR products (bp)** | | | | | | |
| 1 | 132 | 198 | ­- | 98 | 225 | 169 | 176 |
| 2 | 138 | 204 | 164 | 110 | 231 | 175 | 182 |
| 3 | 144 | 210 | 170 | 122 | 237 | 181 | 188 |
| 4 | 150 | 216 | 176 | 134 | 243 | 187 | 194 |
| 5 | 156 | 222 | 182 | 146 | 249 | 193 | 200 |
| 6 | 162 | 228 | 188 | 158 | 255 | 199 | 206 |
| 7 | 168 | 234 | 194 | 170 | 261 | 205 | 212 |
| 8 | 174 | 240 | 200 | 182 | 267 | 211 | 218 |
| 9 | 180 | 246 | 206 |  | 273 | 217 | 224 |
| 10 | 186 | 252 | 212 |  | 279 | 223 | 230 |
| 11 | 192 | 258 | 218 |  | 285 | 229 | 236 |
| 12 | 198 | 264 | 224 |  | 291 | 235 | 242 |
| 13 |  | 270 | 230 |  | 297 | 241 | 248 |
| 14 |  | 276 | 236 |  | 303 | 247 | 254 |
| 15 |  | 282 | 242 |  | 309 | 253 |  |
| 16 |  | 288 | 248 |  | 315 |  |  |
| 17 |  | 294 |  |  | 321 |  |  |
| 18 |  | 300 |  |  | 327 |  |  |
| 19 |  | 306 |  |  | 333 |  |  |
| 20 |  | 312 |  |  | 339 |  |  |
| 21 |  | 318 |  |  | 345 |  |  |
| 22 |  | 324 |  |  |  |  |  |
| 23 |  | 330 |  |  |  |  |  |
| 24 |  | 336 |  |  |  |  |  |
| 25 |  | 342 |  |  |  |  |  |
| 26 |  | 348 |  |  |  |  |  |
| 27 |  | 354 |  |  |  |  |  |
| 28 |  | 360 |  |  |  |  |  |
| 29 |  | 366 |  |  |  |  |  |
| 30 |  | 372 |  |  |  |  |  |
| 31 |  | 378 |  |  |  |  |  |
| 32 |  | 384 |  |  |  |  |  |
| 33 |  | 390 |  |  |  |  |  |
| 34 |  | 396 |  |  |  |  |  |
| 0 |  |  |  |  | 219 |  |  |

Table S2.

List of strains submitted for whole genome sequencing as well as the corresponding serotype, biotype, MLST type, MLVA profile, SNP Address and 5-SNP cluster as well as geographical distribution (DHB) of yersiniosis cases.

| **Strain ID** | **Serotype** | **Biotype** | **MLST** | **MLVA** | **SNP Address** | **5-SNP Cluster** | **DHB** |
| --- | --- | --- | --- | --- | --- | --- | --- |
| 15ER3835 | O:9 | 2 | 12 | 5-8-7-4-4-7-8 | 1.1.1.3.3.67.71 | 1.1.1.3.3.67 | Waikato |
| 15ER3864 | O:9 | 2 | 12 | 5-7-5-4-4-7-7 | 1.1.1.44.54.65.67 | 1.1.1.44.54.65 | Tairawhiti |
| 15ER3866 | O:9 | 2 | 12 | 4-8-8-4-4-6-8 | 1.1.1.45.55.66.68 | 1.1.1.45.55.66 | Canterbury |
| 15ER3878 | O:9 | 2 | 12 | 4-8-7-4-4-6-8 | 1.1.1.3.57.69.73 | 1.1.1.3.57.69 | Capital and Coast |
| 15ER3906 | O:9 | 2 | 12 | 4-9-7-5-4-6-9 | 1.1.1.3.18.18.69 | 1.1.1.3.18.18 | Auckland |
| 15ER3972 | O:9 | 2 | 12 | 4-8-8-4-4-6-8 | 1.1.1.45.55.66.68 | 1.1.1.45.55.66 | Capital and Coast |
| 15ER3974 | O:9 | 2 | 12 | 4-9-7-5-4-6-9 | 1.1.1.3.18.18.64 | 1.1.1.3.18.18 | Capital and Coast |
| 15ER3975 | O:9 | 2 | 12 | 4-9-7-4-4-6-9 | 1.1.1.3.18.18.70 | 1.1.1.3.18.18 | Hutt Valley |
| 15ER3977 | O:9 | 2 | 12 | 4-9-7-4-4-6-9 | 1.1.1.3.18.18.18 | 1.1.1.3.18.18 | Capital and Coast |
| 15ER3994 | O:9 | 2 | 12 | 4-8-8-4-4-6-8 | 1.1.1.45.55.66.68 | 1.1.1.45.55.66 | Waitemata |
| 15ER4021 | O:9 | 2 | 12 | 4-8-8-4-4-6-8 | 1.1.1.45.55.66.68 | 1.1.1.45.55.66 | West Coast |
| 15ER4046 | O:9 | 2 | 12 | 4-9-7-3-4-6-9 | 1.1.1.3.18.18.18 | 1.1.1.3.18.18 | Hutt Valley |
| 15ER4205 | O:9 | 2 | 12 | 4-9-7-5-4-6-9 | 1.1.1.3.18.18.18 | 1.1.1.3.18.18 | Capital and Coast |
| 16ER1274 | O:9 | 2 | 12 | 4-8-5-4-4-6-8 | 1.1.1.3.8.56.56 | 1.1.1.3.8.56 | Canterbury |
| 16ER1398 | O:9 | 2 | 12 | 4-8-8-4-4-6-8 | 1.1.1.3.4.4.57 | 1.1.1.3.4.4 | Canterbury |
| 16ER1851 | O:9 | 2 | 12 | 4-7-7-4-4-6-7 | 1.1.1.3.32.32.32 | 1.1.1.3.32.32 | Auckland |
| 16ER2037 | O:9 | 2 | 12 | 4-7-8-4-4-6-7 | 1.1.1.3.4.4.33 | 1.1.1.3.4.4 | Auckland |
| 16ER3479 | O:3 | 4 | 18 | 6-7-10-2-3-8-7 | 4.4.4.16.16.16.16 | 4.4.4.16.16.16 | Capital and Coast |
| 16ER3758 | O:9 | 2 | 12 | 4-7-8-4-4-6-7 | 1.1.1.3.4.4.4 | 1.1.1.3.4.4 | West Coast |
| 16ER3762 | O:5,27 | 3 | 14 | 6-14-6-2-6-8-14 | 2.2.3.15.15.15.15 | 2.2.3.15.15.15 | Bay of Plenty |
| 16ER3788 | O:9 | 3 | 12 | 4-8-9-4-4-6-8 | 1.1.1.3.4.53.53 | 1.1.1.3.4.53 | Auckland |
| 16ER3887 | O:9 | 2 | 12 | 4-7-8-4-4-6-7 | 1.1.1.3.4.4.38 | 1.1.1.3.4.4 | Waikato |
| 16ER3919 | O:9 | 2 | 12 | 4-7-8-4-4-6-7 | 1.1.1.3.4.4.7 | 1.1.1.3.4.4 | Bay of Plenty |
| 16ER3942 | O:9 | 2 | 12 | 5-7-7-4-4-7-7 | 1.1.1.3.3.35.35 | 1.1.1.3.3.35 | Canterbury |
| 16ER3959 | O:9 | 3 | 12 | 4-10-5-3-3-6-10 | 1.1.1.2.2.2.2 | 1.1.1.2.2.2 | Bay of Plenty |
| 16ER3961 | O:9 | 3 | 12 | 5-7-8-4-4-7-7 | 1.1.1.3.24.24.24 | 1.1.1.3.24.24 | Bay of Plenty |
| 16ER3998 | O:9 | 3 | 12 | 4-10-5-3-3-6-10 | 1.1.1.2.2.2.2 | 1.1.1.2.2.2 | Bay of Plenty |
| 16ER4033 | O:9 | 2 | 12 | 4-10-5-3-3-6-10 | 1.1.1.2.2.2.2 | 1.1.1.2.2.2 | Bay of Plenty |
| 16ER4035 | O:9 | 2 | 12 | 8-9-5-4-4-10-9 | 1.1.1.3.8.63.63 | 1.1.1.3.8.63 | Bay of Plenty |
| 16ER4036 | O:9 | 2 | 12 | 4-10-5-3-3-6-10 | 1.1.1.2.2.2.22 | 1.1.1.2.2.2 | Bay of Plenty |
| 16ER4045 | O:9 | 2 | 12 | 5-7-7-2-4-7-7 | 1.1.1.3.3.45.45 | 1.1.1.3.3.45 | Southern |
| 16ER4069 | O:9 | 2 | 12 | 4-10-5-3-3-6-10 | 1.1.1.2.2.2.2 | 1.1.1.2.2.2 | Bay of Plenty |
| 16ER4078 | O:9 | 2 | 12 | 4-8-9-4-4-6-8 | 1.1.1.3.4.5.10 | 1.1.1.3.4.5 | Canterbury |
| 16ER4090 | O:9 | 2 | 12 | 4-10-5-3-3-6-10 | 1.1.1.2.2.2.2 | 1.1.1.2.2.2 | Bay of Plenty |
| 16ER4091 | O:9 | 2 | 12 | 4-11-5-3-3-6-11 | 1.1.1.2.2.2.2 | 1.1.1.2.2.2 | Bay of Plenty |
| 16ER4099 | O:9 | 2 | 12 | 4-10-5-3-3-6-10 | 1.1.1.2.2.2.2 | 1.1.1.2.2.2 | Bay of Plenty |
| 16ER4101 | O:9 | 2 | 12 | 4-10-5-3-3-6-10 | 1.1.1.2.2.2.2 | 1.1.1.2.2.2 | Bay of Plenty |
| 16ER4128 | O:9 | 2 | 12 | 4-10-5-3-3-6-10 | 1.1.1.2.2.2.2 | 1.1.1.2.2.2 | Bay of Plenty |
| 16ER4129 | O:9 | 2 | 12 | 5-7-7-4-4-7-7 | 1.1.1.3.3.3.3 | 1.1.1.3.3.3 | Bay of Plenty |
| 16ER4130 | O:9 | 2 | 12 | 4-10-5-3-3-6-10 | 1.1.1.2.2.2.2 | 1.1.1.2.2.2 | Bay of Plenty |
| 16ER4131 | O:9 | 2 | 12 | 4-10-5-3-3-6-10 | 1.1.1.2.2.2.19 | 1.1.1.2.2.2 | Bay of Plenty |
| 16ER4132 | O:9 | 2 | 12 | 4-10-5-3-3-6-10 | 1.1.1.2.2.2.2 | 1.1.1.2.2.2 | Bay of Plenty |
| 16ER4151 | O:9 | 2 | 12 | 3-8-7-4-4-5-8 | 1.1.1.3.6.21.21 | 1.1.1.3.6.21 | Auckland |
| 16ER4152 | O:9 | 2 | 12 | 4-10-5-3-3-6-10 | 1.1.1.2.2.2.2 | 1.1.1.2.2.2 | Bay of Plenty |
| 16ER4167 | O:9 | 2 | 12 | 6-5-8-4-4-8-5 | 1.1.1.20.20.20.20 | 1.1.1.20.20.20 | Bay of Plenty |
| 16ER4171 | O:9 | 2 | 12 | 4-10-5-3-3-6-10 | 1.1.1.2.2.2.37 | 1.1.1.2.2.2 | Bay of Plenty |
| 16ER4172 | O:9 | 2 | 12 | 4-10-5-3-3-6-10 | 1.1.1.2.2.2.2 | 1.1.1.2.2.2 | Bay of Plenty |
| 16ER4202 | O:9 | 2 | 12 | 4-10-5-3-3-6-10 | 1.1.1.2.2.2.2 | 1.1.1.2.2.2 | Bay of Plenty |
| 16ER4230 | O:9 | 2 | 12 | 4-7-8-4-4-6-7 | 1.1.1.3.4.4.13 | 1.1.1.3.4.4 | Bay of Plenty |
| 17ER0296 | O:5,27 | 2 | 14 | 10-9-6-2-4-12-9 | 2.2.8.41.41.41.41 | 2.2.8.41.41.41 | West Coast |
| 17ER0312 | O:9 | 3 | 12 | 4-10-5-3-3-6-10 | 1.1.1.2.2.2.65 | 1.1.1.2.2.2 | Capital and Coast |
| 17ER0387 | O:5,27 | 3 | 14 | 9-8-8-2-5-11-8 | 2.2.2.14.36.47.47 | 2.2.2.14.36.47 | Auckland |
| 17ER0440 | O:9 | 2 | 12 | 4-8-5-4-3-6-8 | 1.1.1.2.42.42.61 | 1.1.1.2.42.42 | Bay of Plenty |
| 17ER0444 | O:5,27 | 2 | 14 | 10-10-6-2-5-12-10 | 2.2.2.14.36.51.51 | 2.2.2.14.36.51 | Northland |
| 17ER0471 | O:9 | 2 | 12 | 4-10-5-3-3-6-10 | 1.1.1.2.2.2.2 | 1.1.1.2.2.2 | Tairawhiti |
| 17ER0614 | O:9 | 2 | 12 | 8-9-5-4-4-10-9 | 1.1.1.3.8.8.8 | 1.1.1.3.8.8 | Bay of Plenty |
| 17ER0621 | O:5,27 | 2 | 14 | 9-9-7-2-5-11-9 | 2.2.2.14.36.36.36 | 2.2.2.14.36.36 | South Canterbury |
| 17ER0642 | O:9 | 2 | 12 | 3-8-8-4-4-5-8 | 1.1.1.3.6.21.34 | 1.1.1.3.6.21 | Hawke's Bay |
| 17ER0684 | O:9 | 2 | 12 | 4-8-7-4-4-6-8 | 1.1.1.3.6.6.6 | 1.1.1.3.6.6 | Southern |
| 17ER0827 | O:9 | 2 | 12 | 4-8-5-4-3-6-8 | 1.1.1.2.42.42.46 | 1.1.1.2.42.42 | Bay of Plenty |
| 17ER0866 | O:5,27 | 3 | 14 | 4-8-5-4-4-6-8 | 2.2.2.14.14.14.14 | 2.2.2.14.14.14 | Canterbury |
| 17ER0929 | O:9 | 3 | 12 | 4-7-8-4-4-6-7 | 1.1.1.3.4.17.17 | 1.1.1.3.4.17 | Capital and Coast |
| 17ER0940 | O:9 | 2 | 12 | 4-9-9-4-4-6-9 | 1.1.1.3.4.5.5 | 1.1.1.3.4.5 | Northland |
| 17ER0966 | O:9 | 3 | 12 | 4-7-8-4-4-6-7 | 1.1.1.3.4.4.39 | 1.1.1.3.4.4 | Taranaki |
| 17ER0967 | O:9 | 2 | 12 | 4-7-8-4-4-6-7 | 1.1.1.3.4.4.40 | 1.1.1.3.4.4 | Lakes |
| 17ER0976 | O:9 | 2 | 12 | 5-7-8-5-4-7-7 | 1.1.1.3.29.29.29 | 1.1.1.3.29.29 | Waitemata |
| 17ER1029 | O:3 | 4 | 18 | 6-7-14-2-3-8-7 | 4.4.4.16.16.16.49 | 4.4.4.16.16.16 | Bay of Plenty |
| 17ER1031 | O:9 | 2 | 12 | 4-8-5-4-3-6-8 | 1.1.1.2.42.42.42 | 1.1.1.2.42.42 | Bay of Plenty |
| 17ER1088 | O:9 | 2 | 12 | 4-10-5-3-3-6-10 | 1.1.1.2.2.2.25 | 1.1.1.2.2.2 | Bay of Plenty |
